# Dasatinib‐induced chylothorax: An unusual presentation of a common adverse event—A case report with literature review

**DOI:** 10.1002/jha2.226

**Published:** 2021-05-18

**Authors:** Theresa Paul, Anil Yousaf Ellahie, Yazan Salah Almohtasib, Urshita Sinha, Halima El Omri

**Affiliations:** ^1^ Department of Internal Medicine Hamad Medical Corporation Doha Qatar; ^2^ Department of Hematology National Centre for Cancer Care and Research Doha Qatar

**Keywords:** chylothorax, Dasatinib, glucose–lipid metabolism, imatinib, leukemia, pleural effusion, tyrosine kinase inhibitors

## Abstract

Tyrosine kinase inhibitors (TKIs) are the key agents for treating CML and BCR–ABL^+^ B‐ALL. Dasatinib is a potent second‐generation TKI. Here, we have discussed the case of a 51‐year‐old gentleman diagnosed with B‐myeloid mixed‐phenotype acute leukemia with t(9;22)(q34.1;q11.2); BCR–ABL1p210, in complete hematological, cytogenetic, and molecular remission, who developed chylothorax. Though pleural effusion is a commonly observed adverse effect of dasatinib therapy, chylothorax is rare. The ability of Dasatinib to inhibit multiple families of tyrosine kinases could be considered the etiology. Discontinuation of the drug resolved the symptom, but pleural effusion recurred once Dasatinib was resumed. Chylothorax induced by Dasatinib is a differential to be kept in mind, owing to the limited number of cases being reported.

## 1 INTRODUCTION

Tyrosine kinase inhibitors (TKIs) are the cornerstone agents for treating CML and BCR–ABL^+^ B‐ALL. Approved in 2006 by the FDA, Dasatinib is a potent second‐generation TKI, used as a first‐line treatment of CML and BCR–ABL^+^ B‐ALL [1]. Pleural effusion is a common adverse effect occurring around 28%–33% of patients on long‐term therapy with Dasatinib [2]. Chylothorax is an extremely unusual presentation with approximately 10 cases of Dasatinib‐related chylothorax reported in the literature. The exact pathophysiology of this particularly rare occurrence remains unclear. Here, we discuss a case of Dasatinib‐induced chylothorax in a 51‐year‐old male patient on Dasatinib as maintenance for BCR–ABL^+^ B‐ALL in clinical remission.

## 2 CASE

A 51‐year‐old gentleman with only known past medical history of essential hypertension presented in June 2019, with severe anemia symptoms of fatigue, exertional shortness of breath, and dizziness. Initial blood works showed remarkable leukocytosis (123.1 × 10^3^/μl), severe macrocytic anemia (Hgb: 5.0 gm/dl), and thrombocytopenia (60 × 10^3^/μl) with approximately 80% circulating blasts with a shift to left, dysgranulopoiesis, and monocytosis.

Bone marrow examination with immunophenotyping and cytogenetic was consistent with acute leukemia favoring B‐myeloid mixed‐phenotype acute leukemia with t(9;22)(q34.1;q11.2); BCR‐ABL1p210. His initial computed tomography (CT) scan at diagnosis showed bilateral axillary and mediastinal lymphadenopathy, and both lungs were clear with no pleural effusion.

The disease was considered high risk, and we planned to start him on Hyper‐CVAD protocol (two cycles), Dasatinib 140 mg oral daily, and allogeneic transplant.

He was started on induction treatment with Hyper‐CVAD (hyperfractionated cyclophosphamide, vincristine, doxorubicin, and dexamethasone, alternating with high‐dose methotrexate, cytarabine, and intrathecal methotrexate and cytarabine), side by side with TKI, Dasatinib 140 mg oral daily. He received a total of three cycles of Hyper‐CVAD

Evaluation after two cycles confirmed complete hematological, cytogenetic, and molecular remission. The patient had no matching sibling donor, and he continued a third cycle of Hyper‐CVAD and Dasatinib with molecular follow‐up every 3 months. The treatment was well‐tolerated, and he remained in hematological and molecular remission.

**FIGURE 1:**
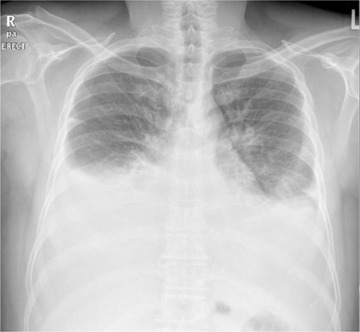
Chest X‐ray on presentation showing bilateral pleural effusion

**FIGURE 2:**
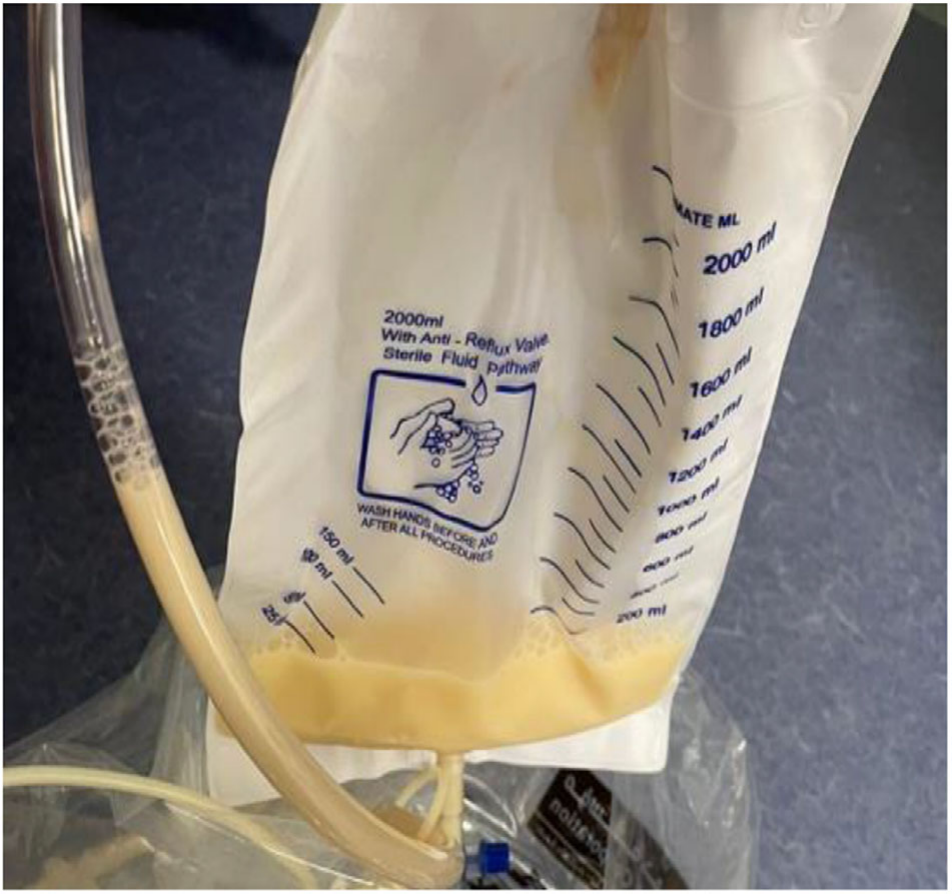
Chylous fluid drained freely after thoracocentesis

In February 2021, around 20 months after starting Dasatinib, he presented to the emergency department with shortness of breath, fever, and cough. Physical examination revealed reduced breathing sounds bilaterally with stony dullness up to midzone. Chest X‐ray showed bilateral moderate pleural effusion (Figure 1), drained under ultrasound guidance to reveal a surprisingly looking milky fluid (Figure 2).

**FIGURE 3:**
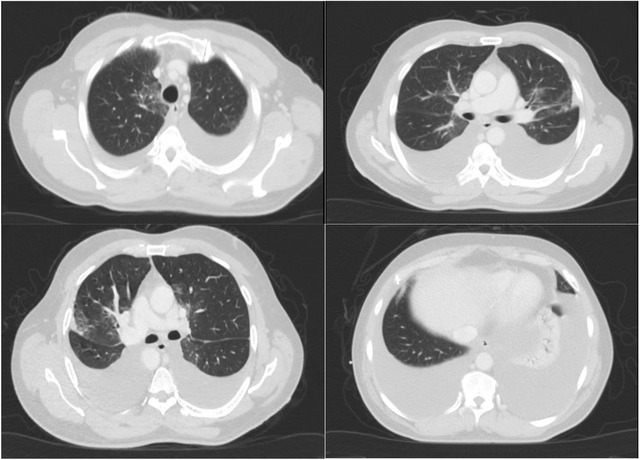
CT thorax showing significant bilateral pleural effusion with associated collapse/ consolidation of the bases of both lungs and areas of ground‐glass appearances and incomplete consolidations

Biochemical analysis of the pleural fluid disclosed lymphocytic exudative effusion with a triglyceride level of 15.2 mmol/L (1346.3 mg/dl). Thorough blood investigations and pleural fluid cytology results were noncontributory toward the etiology of chylothorax. The patient but had new‐onset hyperlipemia and hypertriglyceridemia in the blood, evidenced by total cholesterol of 7.07 mmol/L (273 mg/dl) and triglyceride levels of 7.74 mmol/L (685 mg/dl). Flow cytometry analysis on the pleural fluid did not demonstrate any clonal B‐cell population. Furthermore, CT scan of the thorax failed to point to an etiology for the pleural fluid's chylous nature or any features of residual disease (Figure 3). Dasatinib was held as the probable cause for the pleural effusion, and he was started on prednisolone 30 mg daily for 5 days, with appropriate diuresis. A total of 1160 and 1350 ml of fluid was removed from right and left hemithoraces, respectively. Bone marrow evaluation with immunophenotyping and cytogenetic and molecular analysis confirmed the persistence of complete remission (CR). FDG PET CT was also done to evaluate any possible cause for the unusual effusion; however, results showed only the presence of bilateral pleural effusion.

After resolution of the pleural effusion, the patient was finally discharged home after switching to Imatinib.

## 3 DISCUSSION

The patient in this report presented with bilateral chylothorax. After reviewing detailed history and investigations, no other etiology for the chylothorax was identified other than Dasatinib.

Chylothorax is the accumulation of chyle in the pleural cavity caused by its extravasation into the pleural space. It can be due to obstruction or injury to the thoracic duct or its tributaries or transdiaphragmatic flow from the peritoneal cavity. Pleural fluid is often milky in appearance with triglycerides >1.24 mmol/L (>110 mg/dl) or the presence of chylomicrons [3, 4]. Chylothorax is a lesser common pleural effusion with a broad differential centering around impaired lymphatic drainage, most commonly due to trauma, malignancy, or lymphatic disorders. Despite these numerous etiologies of chylothorax, Dasatinib is the only drug described in the literature associated with such a finding [5].

Dasatinib is a potent TKI with the ability to inhibit multiple tyrosine kinases, including the SRC family of kinases in addition to BCR–ABL1. It is an orally administered small molecule with additional inhibitory activity for c‐KIT, EPHA2, and platelet‐derived growth factor receptor‐beta (PDGFR‐B) [6, 7, 8].

In vitro, Dasatinib was found to be 325‐fold more potent in inhibiting the wild type of BCR–ABL1 compared to Imatinib, making it an effective agent in Imatinib‐resistant BCR–ABL1 mutants [9]. In the two major long‐term follow‐up studies of Dasatinib—DASISION and CA180‐034, exudative pleural effusion occurred in about 30% of patients, occurring anytime from the first year of therapy to up to 6 years into follow‐up [2, 10].

Dasatinib‐induced chylothorax is particularly rare, and its pathophysiology is poorly understood. A few of the proposed mechanisms are related to the pharmacodynamics of the drug itself. Microscopic disruption of the lymphatic channels rather than direct obstruction of the thoracic duct could be considered the etiology of chylous effusion in our patient [5]. The ability of Dasatinib to inhibit the tyrosine kinase PDGFR‐B and the Src family of kinases might be the critical factor in the pathophysiology of the development of chylothorax. PDGFR‐B regulates angiogenesis, lymphangiogenesis, mesangial and vascular smooth cell proliferation, and pericyte recruitment of capillaries, the inhibition of which results in impaired vascular remodeling [9, 11, 12].

Src family of kinases are well expressed on the hematopoietic cells in the lung tissue and mediate the pleural epithelium's vascular permeability and stability [13, 14]. Both these actions are compromised by the ability of Dasatinib to inhibit multiple tyrosine kinases. The pleural fluid's lymphocytic predominance in all the cases also points toward the possibility of an immune‐mediated mechanism for this adverse event [15].

Dasatinib‐induced pleural effusions are managed with various strategies, including temporary interruptions, diuretics, and/or low‐dose steroids and thoracocentesis [16]. Discontinuation of the drug resolved the symptom, and around 70% of the patients had a recurrence of pleural effusion once Dasatinib was resumed. The development of pleural effusion was also found to be dose related [17]. A dose of 100 mg daily was found to have lower pleural effusion incidences than higher doses [18]. The other management strategies included daily dose reduction or, as an alternative option, an on/off treatment with a weekend drug holiday was also found to be effective [19]. With persistence or recurrence of pleural effusion, the patient was challenged with a different TKI instead of Dasatinib such as Imatinib, which was associated with a much lesser pleural effusion incidence [20]. Most of the cases of Dasatinib‐induced chylothorax in the literature were bilateral and ultimately led to the discontinuation of the drug. Reintroduction of Dasatinib despite reducing the dose or short drug holidays resulted in the recurrence of the chylothorax. Dasatinib was later switched to another appropriate TKI, as most strategies failed (Table 1).

**TABLE 1:** Summary of the characteristics of the cases described in literature on Dasatinib‐related chylothorax, including our case

Case	1	2	3	4	5	6	7	8	9	10	11 (our case)
Year	2015	2016	2016	2016	2016	2017	2019	2019	2020	2020	2021
Age	40	71	47	46	49	69	71	63	84	63	51
Gender	Female	Female	Male	Male	Male	Male	Male	Female	Male	Female	Male
Diagnosis	CML	Ph^+^ ALL	CML	CML	CML	CML	CML	CML	CML	CML	Ph^+^ B‐ALL
Response to treatment with Dasatinib	MMR	Unknown	CMR	MMR	CMR	Unknown	MMR	Unknown	Unknown	Unknown	CMR
Duration from start of therapy to development of chylothorax	40 months	2 months	8 months	19 months	30 months	10 months	6 months	48 months	40 months	12 months	20 months
Dose of Dasatinib	50 mg twice a day	140 mg daily	100 mg daily	100 mg daily	100 mg daily	100 mg daily	100 mg daily	100 mg daily	Unknown	100 mg daily	140 mg daily
Laterality of chylothorax	Bilateral	Bilateral	Unilateral, right	Unilateral, left	Bilateral	Bilateral (right > left)	Bilateral (right > left)	Unilateral, right	Bilateral	Unilateral, left	Bilateral
Triglyceride level in fluid (mg/dl)	Right: 263 Left: 536	Right: 625 Left: 328	Unknown	Unknown	Unknown	Right: 405	Right: 226.5	700	507	334	Right: 1346 Left: 1033
Percentage of lymphocytes	Right: 83% Left: 77%	Right: 85% Left: 80%	Unknown	Unknown	Unknown	Right: 90%	Right: 93%	82%	Predominant (% unknown)	Predominant (% unknown)	Right: 82% Left: 80%
Fluid cultures	Negative	Negative	Negative	Negative	Negative	Negative	Negative	Unknown	Unknown	Unknown	Negative
Dasatinib discontinuation	Yes	Yes	No	Yes	Yes	Yes	Yes	Yes	Yes	Yes	Yes
Management	Thoracocentesis, steroids, diuretics	Thoracocentesis, steroids, diuretics	Thoracocentesis, diuretics	Thoracocentesis, diuretics, thoracic duct ligation	Thoracocentesis, diuretics, thoracic duct ligation	Thoracocentesis only	Thoracocentesis only	Thoracocentesis, diuretics, steroids	Thoracocentesis, steroids, diuretics	Thoracocentesis, Steroids	Thoracocentesis, steroids, diuretics
Final therapy	Nilotinib	Unknown	Dasatinib	Imatinib	Dasatinib	Bosutinib	Follow up	Nilotinib	Unknown	Imatinib	Imatinib
Outcome	Improved	Improved	Improved	Improved	Improved	Improved	Improved	Improved	Improved	Improved	Improved

Abbreviations: ALL, acute lymphoblastic leukemia; CML, chronic myeloid leukemia; CMR, complete molecular remission; MMR, major metabolic remission; Ph^+^, Philadelphia chromosome positive.

1. Huang YM, Wang CH, Huang JS, et al. Dasatinib‐related chylothorax. Turk J Haematol. 2015;32(1):68‐72.

2. Ferreiro L, San‐José E, Suárez‐Antelo J, et al. Dasatinib‐induced pleural effusion: chylothorax, an option to consider. Ann Thorac Med. 2016;11(4):289‐293.

3–5. Yang L, Lu N, Jing Y, et al. Chylothorax related with dasatinib in the treatment of chronic myeloid leukemia: report of 3 cases. Zhongguo Shi Yan Xue Ye Xue Za Zhi. 2016;24(5):1348‐1353. Chinese.

6. Baloch ZQ, Abbas SA, Bhatti H, et al. Dasatinib‐induced chylothorax in chronic myeloid leukemia. Proc (Bayl Univ Med Cent). 2017;30(1):71‐73.

7. Chen B, Wu Z, Wang Q, Li W, Cheng D. Dasatinib‐induced chylothorax: report of a case and review of the literature. Invest New Drugs. 2020;38(5):1627‐1632.

8. Al‐abcha A, Iftikhar MH, Abu Rous F, et al. BMJ Case Rep. 2019;12:e231653. https://doi.org/10.1136/bcr2019‐231653.

9. Bogart M, Amoun T, Messano L, Darcey J, Markley DJ. A Rare Case of Chylothorax Secondary to Dasatinib. InD40. Air and Bodily Fluids in the Thoracic Space: Case Reports in Pleural Disease 2020 May (pp. A6712‐A6712). American Thoracic Society.

10. Molina V, Va˜nes S, Castelló C, Chiner E. Quilotóraxespontáneo secundario a dasatinib. Arch Bronconeumol. 2020;56:599‐601.

Dasatinib also impacts glucose–lipid metabolism compared with Imatinib, which could explain the hyperlipidemia this patient developed. Many studies have investigated abnormal glucose–lipid metabolism of nilotinib and ponatinib, significantly associated with increased cardiovascular events and metabolic syndrome. However, the data on Dasatinib are limited. A small retrospective study of 370 patients on Imatinib, Nilotinib, and Dasatinib found the increased onset of hyperglycemia and hypertriglyceridemia in Nilotinib and Dasatinib compared with Imatinib, the mechanism of which is not fully understood or studied. But it could be attributed to the development of insulin resistance. c‐ABL gene, in in vitro studies, was involved in the insulin receptor (IR) signaling pathway, enhancing the IR metabolic pathway [21, 22].

The exudative and chylous nature of the fluid was managed with caution in our patient. With the background of Ph^+^ B‐ALL in CR, a new‐onset chylothorax would warrant detailed workup, as malignancy‐induced thoracic duct obstruction is the leading cause (61% are lymphoproliferative malignancies) and most important differential to be considered in any patient with chylothorax [23].

## 4 CONCLUSION

Though pleural effusion is a commonly observed adverse effect of dasatinib therapy, chylothorax is particularly rare. The ability of Dasatinib to inhibit multiple families of tyrosine kinases could be considered the etiology of this rare occurrence. However, new‐onset chylothorax in a patient on Dasatinib should be managed with caution, not overlooking other common etiologies. Chylothorax induced by Dasatinib may be kept in mind, owing to the limited number of cases being reported. Considering the therapeutic range and advantage of Dasatinib over other TKIs, especially for Imatinib‐intolerant and Imatinib‐resistant BCR–ABL1 mutants, dosage adjustment in addition to other conservative management needs to be ideally tried first before discontinuing the drug or switching to a different agent. Our patient was already on the daily dosage regimen and also on the lower dose. Hence, changing to Imatinib was thought to be ideal, with a short duration of steroids, diuretics, and bilateral thoracocentesis to relieve the symptoms. He was also started on statin for hyperlipidemia developed. The diverse spectrum of metabolic adverse events of Dasatinib is not fully explored. More studies are needed to ascertain the need for regular monitoring for the development of adverse impact of Dasatinib on glucose–lipid metabolism so as to address the cardiovascular risks early.
